# Atypical Late Presentation of Arrhythmogenic Right Ventricular Cardiomyopathy in a Patient With a History of Childhood Cardiac Surgery

**DOI:** 10.7759/cureus.112691

**Published:** 2026-07-15

**Authors:** Adel Kenawi, Hatem Abdelnasser, Ayman El Masri, Isil Ekin Donmez Erkal

**Affiliations:** 1 Cardiology, Tameside and Glossop Integrated Care NHS Foundation Trust, Ashton-under-Lyne, GBR; 2 Acute Medicine, Tameside and Glossop Integrated Care NHS Foundation Trust, Ashton-under-Lyne, GBR; 3 General Medicine, Tameside and Glossop Integrated Care NHS Foundation Trust, Ashton-under-Lyne, GBR

**Keywords:** arrhythmogenic cardiomyopathy, arrhythmogenic right ventricular cardiomyopathy (arvc), cardiac arrythmia, cardiology imaging, cardiothoracic surgery, cardio vascular disease, inherited cardiomyopathy, right ventricular cardiomyopathy, ventricular dysrhythmia

## Abstract

We report a case of a 53-year-old man with a history of childhood cardiac surgery, presenting with dizziness and broad complex tachycardia. Cardiac imaging revealed classical features of arrhythmogenic right ventricular cardiomyopathy (ARVC), including right ventricular dilation, multiple aneurysms, and non-ischemic fibrosis. There is not much information about the details of his childhood operation. This case highlights the diagnostic value of multi-modal imaging and the relevance of the Padua 2020 criteria in identifying arrhythmogenic cardiomyopathy in adults with prior congenital heart disease. This case provides insight into the challenge of distinguishing primary ARVC from remodelling related to previous childhood cardiac surgery, especially when operative details are limited.

## Introduction

Arrhythmogenic right ventricular cardiomyopathy (ARVC) is a form of heritable cardiomyopathy characterised by fibrofatty replacement of right ventricular myocardium, leading to arrhythmias and structural changes. Although often diagnosed in young individuals, late presentations can occur, especially in patients with prior congenital heart disease. Differentiating primary ARVC from post-surgical remodelling can be challenging and necessitates a comprehensive diagnostic approach, including cardiac MRI and adherence to established criteria like the Padua 2020 framework [1].

Arrhythmogenic cardiomyopathy (ACM) describes a clinical entity in which documented or symptomatic arrhythmia occurs together with structural and functional abnormalities of the myocardium. ARVC, previously termed arrhythmogenic right ventricular dysplasia (ARVD), is a recognised form within the ACM spectrum and has distinct implications for diagnosis, treatment and prognosis. It remains under-recognised clinically and is characterised by ventricular arrhythmias arising from the right ventricle, alongside typical pathological changes affecting the ventricular myocardium [1-3]. Pathologically, ARVC is associated with loss of normal myocardium and its replacement by fibrous or fibro-fatty tissue, which can produce a scar-like macroscopic appearance. Older descriptions placed particular emphasis on right ventricular disease at three sites, the inflow tract, outflow tract and apex, termed the “triangle of dysplasia”. Subsequent evidence has broadened this view, showing that the posterolateral left ventricle can be involved and that apical right ventricular disease may not be present at an early stage [4]. In ARVC, scarring of the right ventricular myocardium may first manifest as localised abnormalities in wall motion. As the disease progresses, these changes can spread across the right ventricular free wall, producing more widespread dysfunction and eventual right ventricular dilatation. Tissue replacement is not limited to the right ventricle, as areas of the left ventricle may also be affected, although the septum is often relatively spared [5].

## Case presentation

A 53-year-old physically active man presented to the Emergency Department with early morning dizziness and general malaise. He denied chest pain, palpitations, or shortness of breath. He worked in a physically demanding job and considered himself fit and healthy with no significant past medical history in his records.

**Figure 1:**
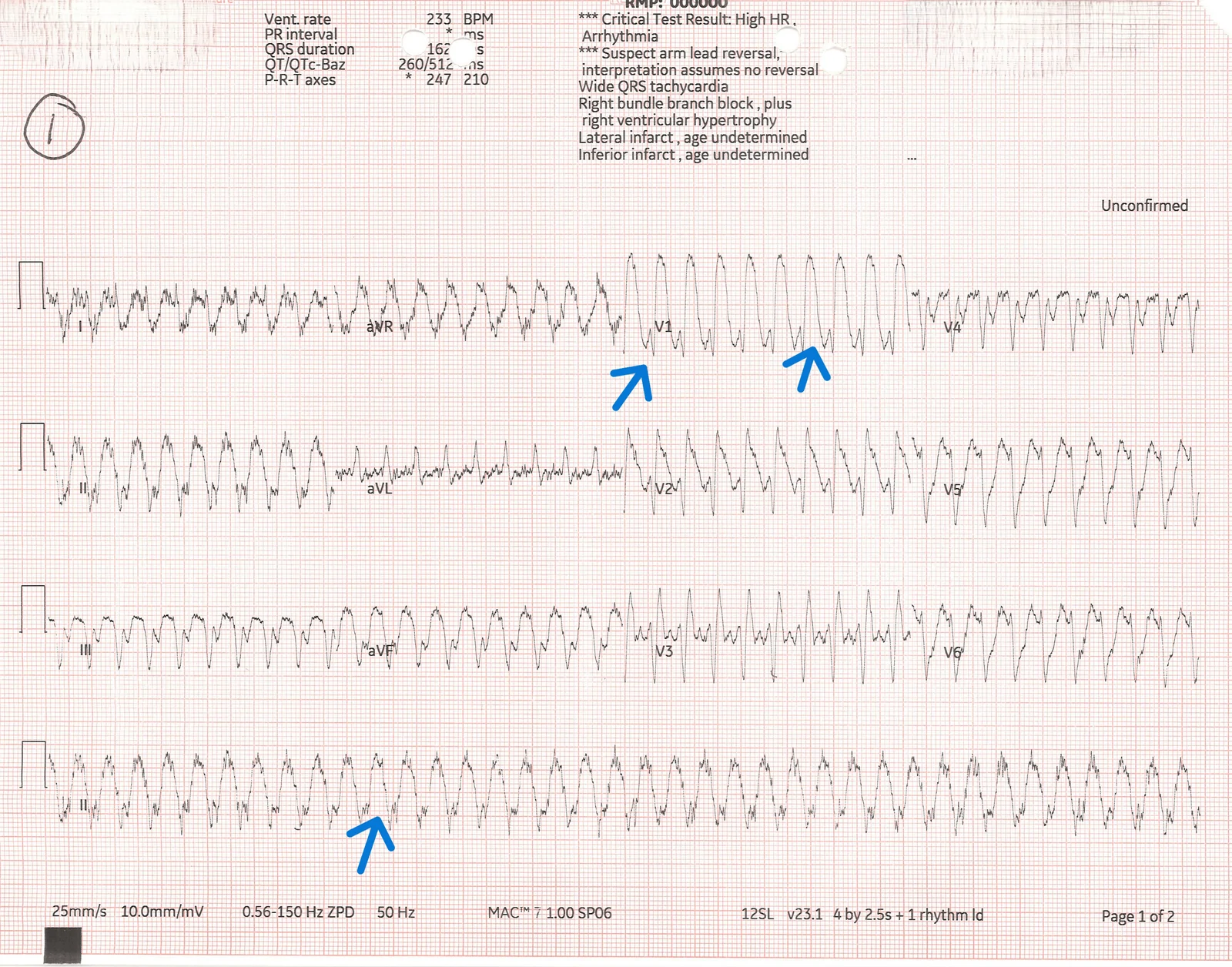
ECG shows regular broad complex tachycardia with heart rate reaching to 233 beats per minute (blue arrows)

His vital signs on presentation were blood pressure 107/79 mmHg (reference values: 100-140/60-90 mmHg), heart rate 233 beats per minute (reference values: 60-100 beats per minute), respiratory rate 18 breaths per minute (reference values: 12-18 breaths per minute), SpO2 98% (reference value: >95%) on room air, and temperature 36.1°C (reference values: 36.5-37.3°C. ECG showed regular broad complex tachycardia (Figure 1).

**Figure 2:**
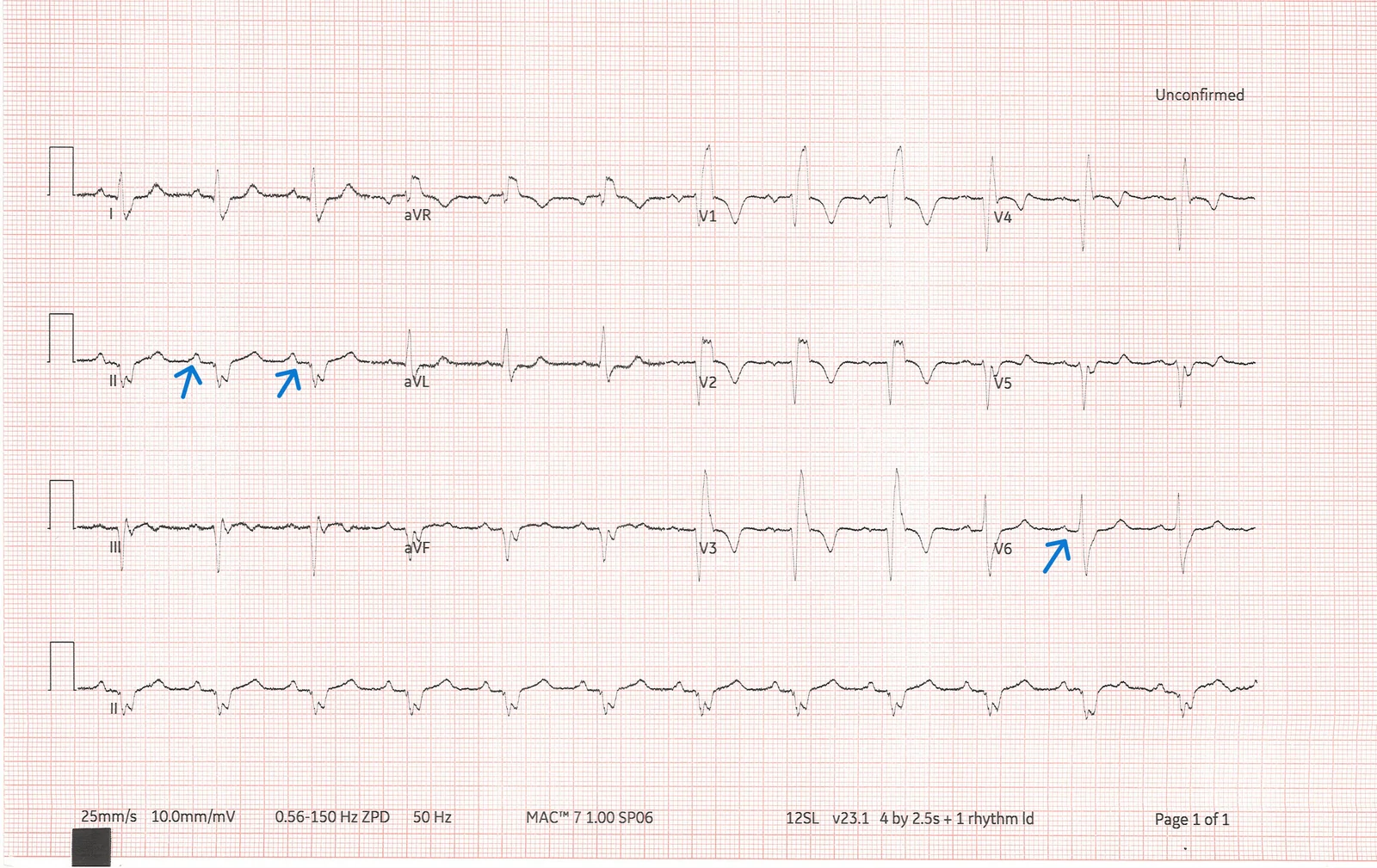
ECG shows restoration of sinus rhythm P-waves before each QRS complex (blue arrows) with right bundle branch block (RBBB) and left axis deviation (LAD). T-wave inversion (TWI) noted in V1- V4.

Subsequently, he reverted to sinus rhythm spontaneously (Figure 2).

Physical examination was unremarkable apart from a prominent midline sternotomy scar. Upon further inquiry, the patient recalled a cardiac surgery in 1976 during childhood but was unaware of the indication or specific procedure. His medical records showed that he had a closed pulmonary valvotomy with no further details about the procedure. His routine bloods showed haemoglobin (Hb) 164 g/L, haematocrit (HCT) 0.47, mean corpuscular volume (MCV) 104 fL, normal arterial blood gas (ABG), renal and liver profile. Thyroid stimulating hormone (TSH) was normal. Troponin I was elevated measuring 117 ng/L. The patient was admitted to the acute coronary care unit (CCU) for telemetry monitoring.

**Figure 3:**
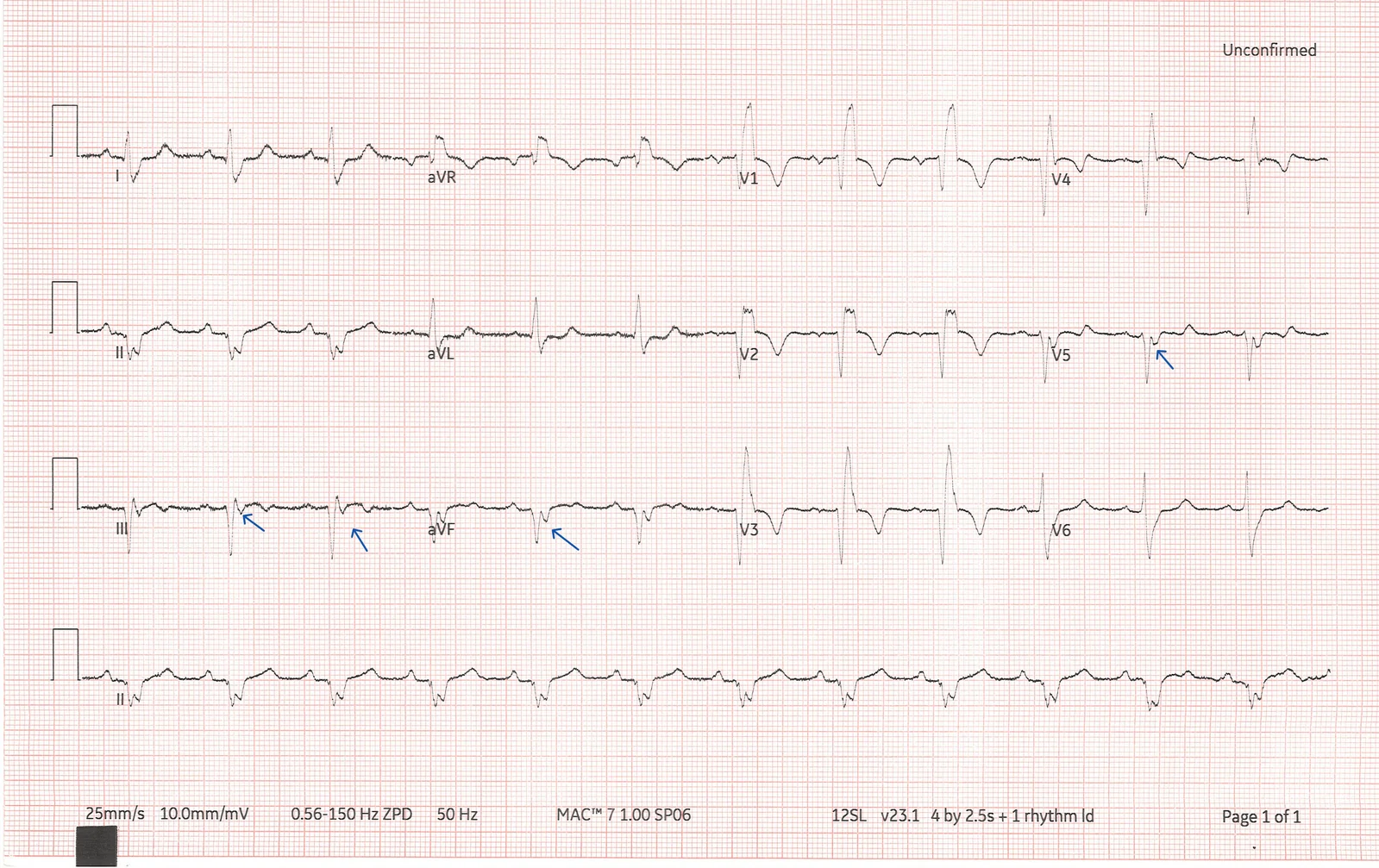
ECG shows sinus rhythm with RBBB morphology and Epsilon waves (blue arrows) RBBB: Right Bundle Branch Block.

**Figure 4:**
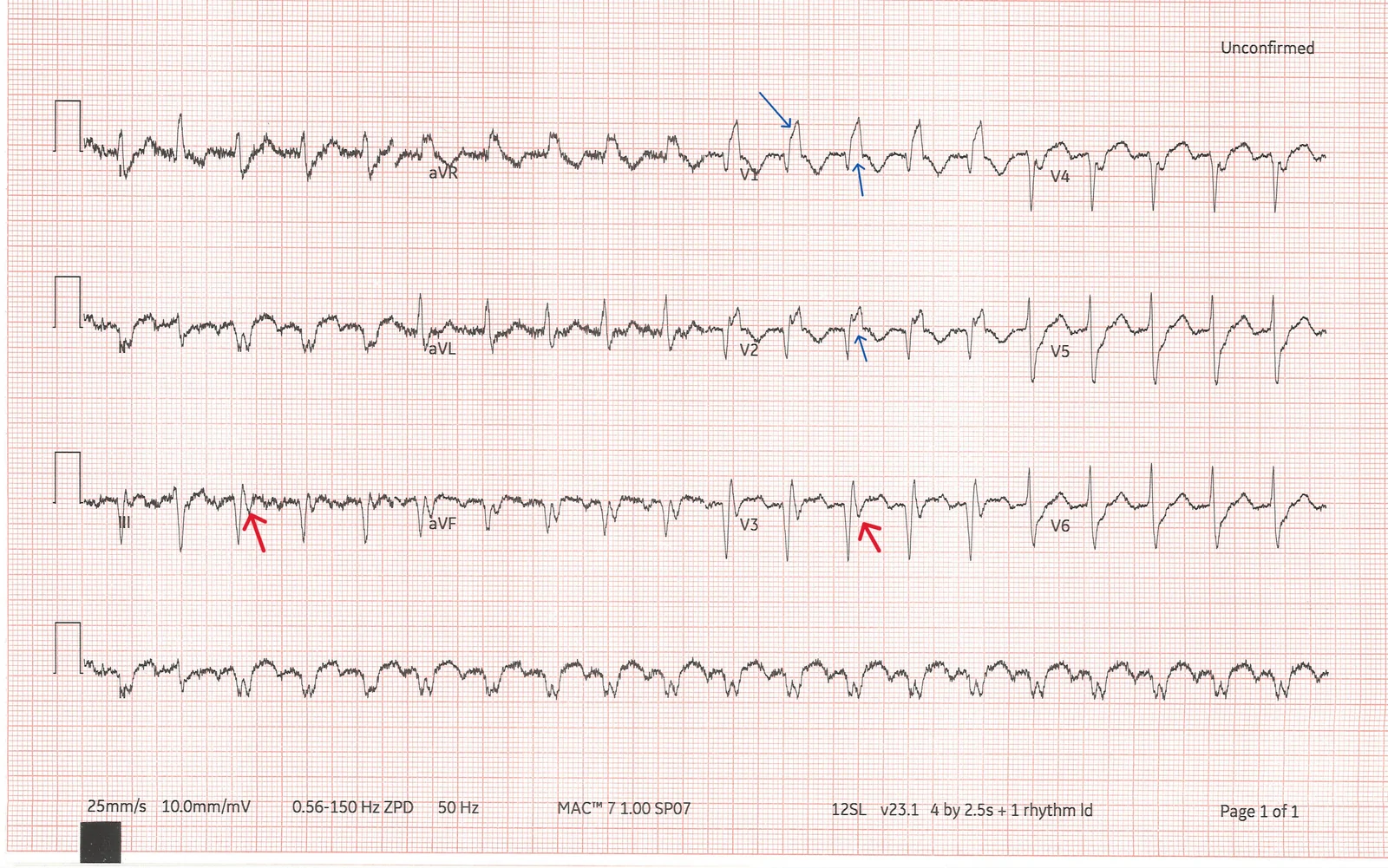
ECG showing regular broad complex tachycardia Demonstrates heart rate of 123 beats per minute, right bundle branch block morphology, Epsilon waves (red arrows) and ST segment elevation (blue arrows).

Serial ECGs revealed frequent episodes of intermittent broad complex tachycardia, atrial flutter alternating with episodes of sinus rhythm, with presence of Epsilon waves (Figures 3, 4). 

**Figure 5:**
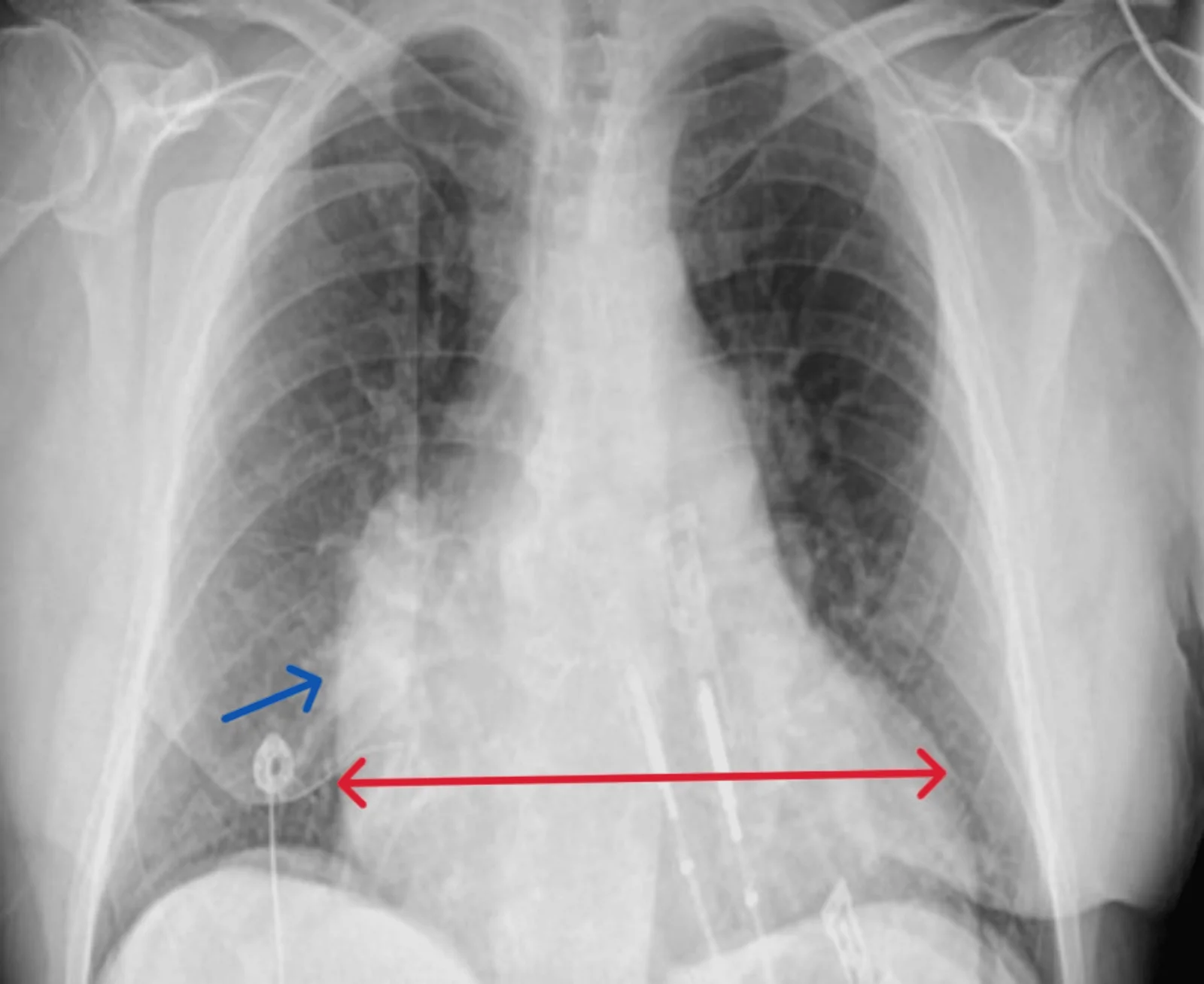
Chest X-ray showing cardiomegaly (red arrow) and enlarged right atrium (blue arrow)

Chest X-ray showed gross cardiomegaly even allowing for the anteroposterior (AP) projection. The lungs were clear with no pulmonary oedema, and no pleural effusion on either side (Figure 5).

Echocardiography showed significantly dilated right ventricular cavity (28.2 cm^2^/m^2^) with significantly impaired overall right ventricular systolic function, and abnormal appearance of the tricuspid valve with significant eccentric tricuspid regurgitation was noted. This was likely severe, however, it was difficult to quantify. There was a high probability of pulmonary hypertension noted by multiple parameters. Overall left ventricular systolic function appeared impaired in the context of the right bundle branch block (RBBB), with an estimated left ventricular ejection fraction (LVEF) of 40%. Mild mitral regurgitation was observed along with significantly dilated right atrium and borderline dilated left atrium (video 1).

**Video 1:** Apical four-chamber view shows dilated right ventricle with extensive trabeculations

Cardiac MRI scan showed normal left ventricular size with localised aneurysmal apical lateral wall and extensive septal and inferior non ischaemic pattern fibrosis seen. Mildly impaired global systolic function was seen with LVEF of 52%. A significantly dilated right ventricle with multiple aneurysmal segments were noted with mild to moderately impaired systolic function (video 1).

**Figure 6:**
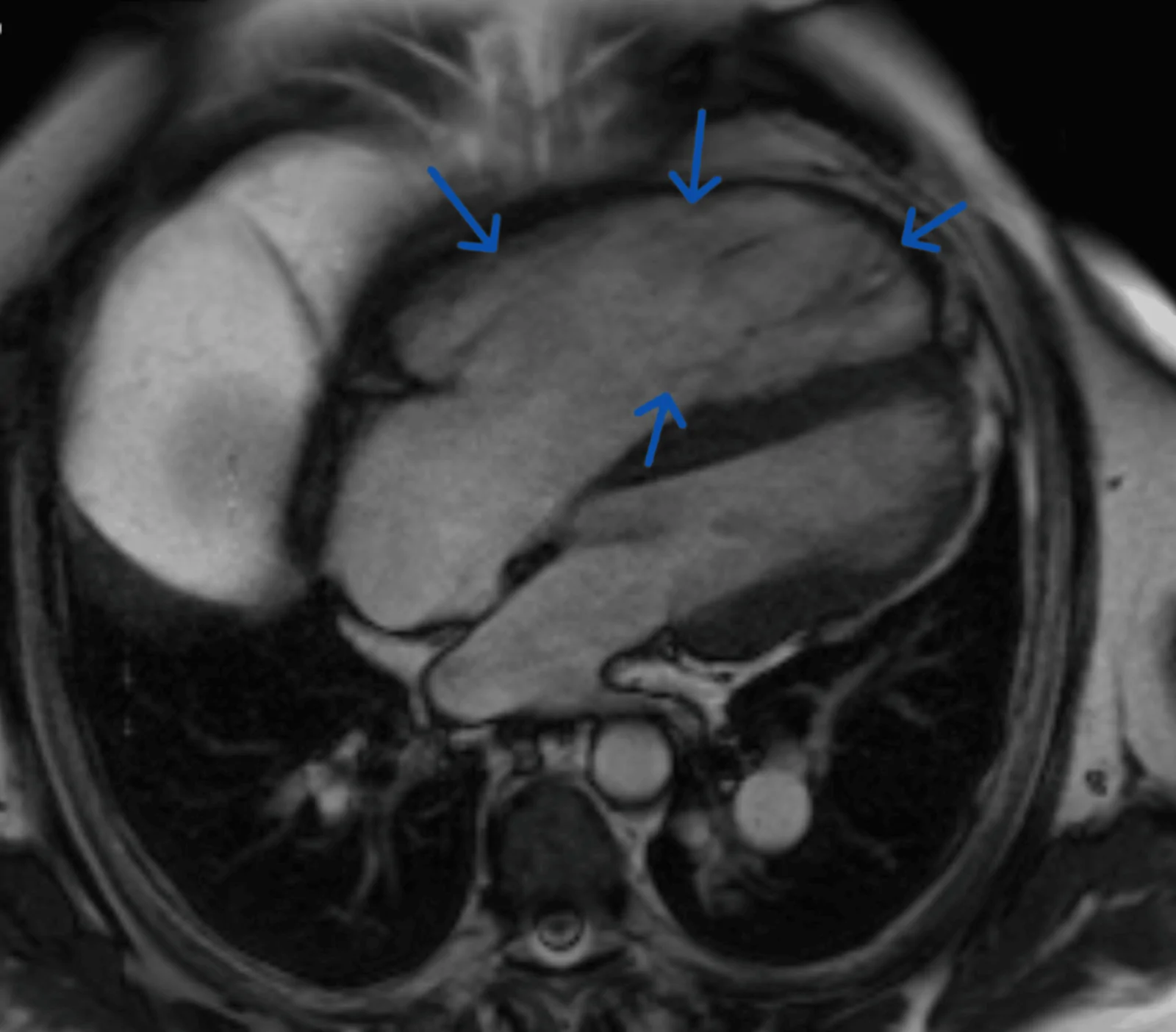
Cardiac MRI scan showing a significantly dilated right ventricle (blue arrows)

Dilated main pulmonary artery with significant pulmonary regurgitation and a right ventricular ejection fraction of 44% was observed. Given the clinical presentation, imaging findings were highly suggestive of ACM (Figure 6 and videos 2, 3).

**Video 2:** Cardiac MRI scan showing significantly dilated RV with mild to moderately impaired systolic function Right ventricular (RV) ejection fraction of 44%.

**Video 3:** Cardiac MRI scan showing a severely dilated pulmonary artery

Diagnosis

Based on the clinical presentation and imaging, the findings were consistent with definite biventricular ACM, fulfilling multiple Padua 2020 criteria, namely major morpho-functional and tissue-characterisation abnormalities, supportive repolarisation and depolarisation abnormalities, ventricular arrhythmia in the form of broad complex tachycardia, a normal left ventricular size with localised aneurysmal apical lateral wall and extensive septal and inferior non ischaemic pattern fibrosis, and global left ventricular systolic dysfunction. The history of childhood heart surgery may represent either an unrepaired or palliated congenital anomaly contributing to structural remodelling, although the exact historical diagnosis remains unclear. The raised troponin was considered secondary to the tachyarrhythmia, likely representing type 2 myocardial infarction.

Management

The patient was admitted to acute cardiac unit (ACU) and loaded with amiodarone initially to control his arrhythmia episodes, which eventually improved. He was also commenced on heart failure treatment in the form of bisoprolol, ramipril, dapagliflozin, and anticoagulated with edoxaban. Amiodarone was discontinued and he didn't have any further episodes of ventricular tachycardia or any significant arrhythmia. 

The case was discussed in a multidisciplinary meeting, including an electrophysiologist along with an inherited cardiac conditions specialist and cardiomyopathy teams. Given the high arrhythmic burden and structural abnormalities, the patient was transferred to a highly specialized tertiary cardiac centre for further work-up and risk stratification; including advanced imaging, electrophysiological studies and consideration of an implantable cardiovertor-defibrillator (ICD) device.

## Discussion

ARVC is an inherited myocardial disease primarily affecting the right ventricle and is characterised by the gradual replacement of myocytes by adipose and fibrous tissue. It affects young people and may cause sudden death, especially during athletic activity [1,4]. Current estimates suggest that ARVC affects around one in 5,000 individuals. However, reported prevalence may increase as clinicians become more familiar with the condition and diagnostic recognition improves [1]. The usual inheritance pattern in ARVC is autosomal dominant, but the condition shows variable penetrance and incomplete expression [1]. Initially, only small areas of the right ventricle may be affected, but the condition may progress over time, becoming more widespread and even involving the left ventricle. In fact, some forms only affect the left ventricle.

As ARVC is a disease of myocardial structure, diagnostic assessment must demonstrate either functional impairment or features consistent with fibrofatty myocardial replacement. A recognised arrhythmic manifestation is ventricular tachycardia with left bundle branch block morphology, particularly in relation to exercise. Since sudden cardiac death is the complication of greatest concern, risk stratification is central to management. Genetic testing is also valuable, as it may identify individuals at risk before a severe first presentation such as cardiac arrest [5].

ARVC most commonly presents from adolescence to early adulthood, although childhood cases are increasingly recognised. Family history is important in assessment and forms part of the diagnostic criteria. However, clinical manifestations can be difficult to predict because penetrance and phenotypic expression vary between individuals [6].

The 2020 Padua criteria expanded the diagnostic framework for ACM by recognising left ventricular disease and emphasising the role of cardiac magnetic resonance imaging. The criteria introduced significant improvements over the 2010 international task force criteria [5]. The criteria are organised into six domains: structural/function, tissue characterisation, repolarisation, depolarisation/conduction, arrhythmias, and family history. Major and minor criteria are included within each domain. Diagnosis requires either two major criteria, one major and two minor criteria, or four minor criteria from separate domains, with at least one criterion from the structural/function or tissue characterisation categories [5,6].

Late-onset presentation after the fifth decade, does not suggest a benign disease course [7]. Symptoms usually emerge between the second and fifth decades of life and are commonly linked to ventricular arrhythmias [8].

Genetics and family screening

Family screening should be offered to first-degree relatives of patients with ARVC, usually starting around age 10 or at the onset of puberty [1]. Familial involvement is common in ARVC, and around one-third of first-degree relatives of an affected proband may develop clinically evident disease. This risk appears to be highest among siblings compared with parents or offspring [9]. Screening should involve both clinical assessment and genetic testing. 

Once ARVC is identified in a proband, first-degree relatives should be followed longitudinally, as disease may become apparent over time. In a cohort from two arrhythmia referral centers, 35% of first-degree relatives were diagnosed with ARVC across nearly seven years of follow-up [9]. In a four-year follow-up study, 30% of relatives demonstrated progression, mainly in the form of electrical abnormalities rather than structural disease [10]. Taken together, these findings support baseline screening of relatives with ECG and echocardiography, with continued follow-up thereafter. Particular emphasis should be placed on identifying electrical manifestations of disease through 12-lead, ambulatory, and exercise ECG [10,11].

More sensitive echocardiographic markers may help detect early myocardial dysfunction during family screening. In first-degree relatives of probands, abnormal right ventricular deformation, assessed using speckle tracking in the apical four-chamber view, was associated with an approximately 10-fold higher risk of disease progression over 3.7 years, even when conventional right ventricular echocardiographic appearances were normal [12,13].

Alongside the genetic component of ARVC, other proposed mechanisms include dysontogenetic, degenerative, infectious or inflammatory, apoptotic, and myocyte trans-differentiation processes. These mechanisms have been suggested either as possible contributors to disease development or as environmental influences that may facilitate gene expression [1].

Clinical presentation

Right ventricular dysplasia can have a varied initial presentation, with serious cardiovascular symptoms and complications occurring frequently among affected patients [14]. ARVC typically occurs in young adult men. Symptoms are usually related to underlying ventricular arrhythmia and heart failure. ARVC should remain a diagnostic consideration in younger patients with syncope, ventricular tachycardia or cardiac arrest, and in adults with congestive heart failure [1,14]. In the U.S., ARVC is estimated to contribute to around 5% of sudden cardiac deaths among people younger than 65 [1].

Diagnosis

The Padua criteria apply a two-step approach to the diagnosis of ACM by assessing major and minor criteria involving the left ventricle, the right ventricle, or both. Based on these findings, ACM is classified into three phenotypes: classic ARVC, biventricular disease, or arrhythmogenic left ventricular cardiomyopathy (ALVC).

Arrhythmogenic left ventricular cardiomyopathy (ALVC)

In ALVC, major criteria include structural myocardial abnormalities demonstrated by late gadolinium enhancement with a stria pattern affecting more than one Bull’s eye segment of the left ventricular free wall, septum, or both [13]. A major genetic criterion is met when a known ACM-causative mutation is identified in the patient or when a first-degree relative satisfies diagnostic criteria or has a confirmed diagnosis following surgery or autopsy. Minor criteria include regional left ventricular hypokinesia or akinesia, global left ventricular systolic dysfunction with or without dilatation, low-voltage QRS complexes in the limb leads, inverted T waves in leads V4 to V6, and ventricular arrhythmias such as sustained or non-sustained ventricular tachycardia or ventricular extrasystoles. Minor genetic and family history criteria include ACM in a second-degree relative, suspected ACM-related sudden death in a first-degree relative under 35 years of age, or a first-degree relative with ACM who does not meet full diagnostic criteria [13,15].

Arrhythmogenic right ventricular cardiomyopathy (ARVC)

Major criteria for ARVC include regional right ventricular dyskinesia, akinesia, or bulging in combination with global right ventricular dilatation or systolic dysfunction identified on cardiac imaging. Additional major criteria include transmural late gadolinium enhancement with a stria pattern in more than one right ventricular region or fibrous myocardial replacement, inverted T waves in leads V1 to V3 in the absence of complete RBBB, frequent ventricular extrasystoles exceeding 500 in 24 hours, sustained or non-sustained ventricular tachycardia with left bundle branch block morphology, and the presence of an ACM-causative mutation or an affected first-degree relative. Minor criteria include less extensive structural abnormalities such as right ventricular free wall aneurysms or regional dyskinesia, repolarisation abnormalities with inverted T waves in leads V1 to V2 without RBBB or in leads V1 to V4 with complete RBBB, depolarisation abnormalities such as epsilon waves or prolonged terminal activation duration, ventricular arrhythmias with a right ventricular outflow tract pattern, and relevant but less definitive genetic or family history findings [13,15].

Interpretation

Diagnosis of ALVC requires the presence of a genetic mutation in addition to phenotypic features. The diagnosis of ARVC can fall into three categories: Definite ARVC: two major or one major plus two minor or four minor right ventricular diagnostic criteria in different categories. Borderline ARVC: one major plus one minor or three minor criteria under different categories. Possible ARVC: one major or two minor criteria. Patients with biventricular ACM fulfil the criteria for both left and right ventricular phenotype [7,13,15]. Our patient fulfilled major morpho-functional and tissue-characterisation criteria. The described multiple right ventricular aneurysmal segments provided additional regional structural support but, if considered alone, would correspond to a minor right ventricular morpho-functional criterion. Minor criteria included T-wave inversion in V1-V4 in the presence of RBBB, epsilon waves, and broad complex tachycardia. Overall, the findings supported a diagnosis of definite biventricular ACM.

Management of patients with ARVC

To reduce the risk of adrenergically mediated arrhythmias, beta-adrenergic blockers are a reasonable treatment option in these patients [1]. If symptoms or documented arrhythmias, such as non-sustained ventricular tachycardia (NSVT), ventricular tachycardia (VT), or frequent complex premature ventricular contractions (PVCs), develop, treatment should be initiated with beta-blockers, or an Implantable Cardioverter-Defibrillator (ICD) in some cases if indicated, together with additional medical therapy to prevent ICD shocks. Amiodarone has also been used effectively, either alone or in combination with other agents, to prevent recurrent arrhythmias. In patients who are intolerant of, or unresponsive to, antiarrhythmic drugs, radiofrequency ablation of the arrhythmogenic focus may be considered, although its success is often limited. For patients at increased risk of sudden cardiac death, ICD implantation is recommended [1].

## Conclusions

ARVC is an inherited myocardial disease primarily affecting the right ventricle but can also affect the left ventricle. The disease can present in adulthood, even decades after presumed childhood repair of congenital heart disease. Cardiac MRI is crucial for identifying right ventricular structural and fibrotic abnormalities and for supporting diagnosis using the Padua 2020 criteria. Unexplained broad complex tachycardia or unexplained cardiac syncope in a fit adult should raise suspicion for underlying cardiomyopathy. Multidisciplinary management and referral to inherited cardiac disease clinics are key in both treatment and family risk assessment.
